# Exploring the Domestic Abuse Narratives of Trans and Nonbinary People and the Role of Cisgenderism in Identity Abuse, Misgendering, and Pathologizing

**DOI:** 10.1177/1077801220971368

**Published:** 2020-12-02

**Authors:** Michaela M. Rogers

**Affiliations:** 1 University of Sheffield, UK

**Keywords:** cisgenderism, domestic abuse, gender identity, microaggressions, nonbinary, trans

## Abstract

Drawing on data from two empirical studies, this article employs cisgenderism as a conceptual tool to explore trans people’s experiences of domestic violence and abuse (DVA). Distinct modes of cisgenderism are analyzed. These are identity abuse, microaggressions, misgendering, and pathologizing practices. Qualitative data were collected via semistructured interviews (*n* = 24). Two inclusion criteria were used for this secondary analysis requiring participants to self-identify as trans or nonbinary and have experience of DVA. The findings illuminate the extent of cisgenderism as underpinning experiences of DVA. The article ends with a call for further theoretical and empirical research in this regard.

## Introduction

Across the Global North, trans and nonbinary people have become central to contemporary discussions of human rights. Yet, despite increased recognition and acceptance, it is frequently reported that trans and nonbinary people remain vulnerable to higher levels of abuse within public and private contexts (European Union Agency for Fundamental Rights, 2015; Grant et al., 2011). Consistent with previous trans/gender research (Beemyn & Rankin, 2011), throughout this article, the term “trans” is employed as an umbrella term to describe a person whose self-identification in relation to gender is different to that which was assigned at birth. This includes a wide range of identities including: trans man, trans woman, transsexual woman, transsexual man, MtF, FtM, a woman or man with a transgender history (Bachmann & Gooch, 2018). “Nonbinary” is also used as a catchall and this incorporates (but is not limited to) the following: genderqueer, genderfluid, agender, bi-gender, pangender, androgynous, androgyne, neutrois, and other identities that describe a person whose gender does not conform with the man/woman binary (Bachmann & Gooch, 2018). As such, in this article, a multiplicity of identities that sit across, along, or outside of a gender spectrum are acknowledged to avoid homogenizing or delimiting the terms trans and nonbinary as ways to indicate gender diversity. Serano’s (2016) philosophical position is adopted which advocates that experience of gender identity is personal and should not be reduced to physical presentation or a set of socially defined characteristics. Similarly, the identities listed are not exhaustive, and it is important to acknowledge the complexity of trans identities (Dargie et al., 2014). Another term increasingly found within the field of gender studies is “cisgender,” or “cis,” which signposts a person whose experience of gender identity remains aligned to that which was assigned at birth (Schilt & Westbrook, 2009).

It is helpful to set out the ontological position adopted in this article as one in which gender is considered to bally constructed and, as such, can be subject to different paradigms in which gender is a form of identification or an aspect of positionality (Rogers & Ahmed, 2017). It may also be conceived at a systemic and structural level as a form of social categorization or organizing device which classifies and orders people according to their material bodies and behaviors (Monro, 2007; Rogers & Ahmed, 2017). There is a plethora of work which explores the sex/gender distinction within a social constructionist ontology (Fausto-Sterling, 2000). Even within a social constructionist framework, however, challenges can be found within scholarship in terms of partitioning accounts of sex and gender. These are frequently seen as interconnected, but simultaneously, this relation is acknowledged to be unstable due to the contingent, situated, and dynamic nature of gender (see Ekins & King, 2006; Rogers, 2020; Rogers & Ahmed, 2017). Despite the wide-ranging body of work that examines gender as a social construct, much of this literature polarizes gender and reduces it to a binary concept of man/masculine and woman/feminine. This marginalizes or invisibilizes trans and nonbinary people who do not ascribe to a binary gender identifier (Biblarz & Savci, 2010).

The settings where trans and nonbinary people experience marginalization and abuse are of both a private and public nature (FRA, 2015; Grant et al., 2011). Within private domains, there is an emerging evidence-base that explores the scale and nature of domestic violence and abuse (DVA), including both intimate partner violence (IPV), and family violence (FV), in trans people’s relationships. In this article, the following definition is in operation in which DVA is a term that seeks to be all-encompassing and incorporates:

Any incident or pattern of incidents of controlling, coercive, threatening behaviour, violence or abuse between those aged 16 or over who are or have been intimate partners or family members regardless of gender or sexuality. (Home Office, 2018, para 1)

This broad definition clearly captures both IPV and FV. Determining prevalence for trans people can be problematic due to the ways in which they are frequently subsumed into the lesbian, gay, bisexual, and trans (LGBT) umbrella (Biblarz & Savci, 2010; Rogers, 2016b, 2017a). This results in the neglect of the heterogeneity and particularity of trans people’s experiences. Further problems in determining prevalence result from varying definitions and measures of violence and methods for operationalizing these definitions, inconsistent reporting or recording, and small or commonly used convenience samples. Despite these methodological challenges, there is a growing body of work suggesting that the scale of IPV and FV among trans communities is at a rate similar to, or higher, than that for cisgender people (James et al., 2016; Langenderfer-Magruder et al., 2016; S. E. Valentine et al., 2017). Importantly, S. E. Valentine et al.’s (2017) study suggests differential risk of DVA within LGBT communities with increased rates of exposure to abuse reported by trans individuals. There is also a growing body of qualitative research which adds rich detail to the picture of trans people’s experiences of DVA (see Brown, 2011; Roch et al., 2010; Rogers, 2016a, 2016b, 2017a).

Data collected in the United States leads the way in portraying prevalence in the Global North, as this is collected relatively frequently as opposed to other countries. For example, in 2015, the U.S. Transgender Survey gathered 27,715 responses from all 50 states. Almost one-quarter (24%) of respondents had experienced severe physical violence by an intimate partner, compared to 18% of the general U.S. population (James et al., 2016). More than half (54%) experienced IPV, including acts of physical violence and coercive control (James et al., 2016). In other parts of the Global North, prevalence data on DVA within LGBT communities are not routinely collected. Despite this, in Australia, an evidence review was undertaken by Campo and Tayton (2015) who found that people who identify within the LGBT umbrella experience DVA at similar rates as those who identify as heterosexual; for instance, one in four women will experience DVA at some point in their lifetime. It was not possible to discern the trans perspective from their report. An earlier survey in Australia found considerable levels of DVA reported by the LGBT-identified respondents (32.7%, total *n* = 308) (SSDVIWG, 2006). However, only two of the sample identified as trans. Campo and Tayton also note how cisgenderism and heterosexism (discrimination or prejudice against homosexuals) intensifies the experience of and outcomes of DVA for LGBT people.

A similar situation is found in Europe as data on DVA and trans communities is variable and patchy. Where data exist, it is often on a localized or small-scale basis, but yields important messages. For example, a small-scale survey (*n* = 71) completed in the United Kingdom found that almost half of respondents (46%) claimed that they had previously experienced transphobic IPV (Scottish Transgender Alliance, 2008). Another study found that 80% of respondents had experienced some form of IPV from a current or former partner (Roch et al., 2010). The Fundamental Rights Agency (FRA) (2015) conducted a larger study including a survey of 6,771 trans-identified people across 28 European member states finding that respondents reported a high level of violence, hate-motivated attacks, and harassment as one in three trans respondents (34%) experienced violence or was threatened with violence in the 5 years preceding the survey. A limitation of this study, however, was that DVA prevalence was not explicitly reported, albeit for those incidents occurring in the home this was mostly within the context of a personal relationship.

## Understanding Cisgenderism

The concept of cisgenderism advances a framework that can help to articulate trans people’s experiences of DVA in micro-level settings (that is, within and across personal relationships) as well as in relation to macro-level influences (for example, social and cultural norms). Cisgenderism integrates ideas of gender normativity. Gender normativity provides a way of understanding how social constructions of binary gender operate as the norm, and any departure from this is rendered abnormal (Blumer et al., 2013; Stryker & Aizura, 2013). A complex set of social norms underpin gender normativity and, at a fundamental level, these promote ideas about cisgender identities as natural and fixed, as well as heterosexual marriage and procreation between a cisgender man and a cisgender woman as the norm. As such, a paradigm of gender which embeds cisgenderism similarly invokes fixed notions of binary gender, gender identities, practices, and bodies. A cisgenderist paradigm promotes the belief that all people are expected to identify within the confines of the gender binary; subsequently, people are judged and accepted on whether they do or do not comply. By this logic, individuals whose self-identified gender differs from that which was assigned at birth, and for those whose gender practices and presentation does not align with dominant gender norms, are considered to be invalid or unnatural (Ansara & Hegarty, 2011, 2014).

Early scholarship on cisgenderism was narrowly tied to the trans/cisgender dichotomy. This is unhelpful, however, as it suggests that trans is reducible to the opposite of cis when it encompasses a wide range of identities that may align with the gender binary, adopt trans as a gender identifier, reject the trans label altogether, or adopt a nonbinary/queer identity (Ansara & Hegarty, 2014). In addition, this earlier scholarship located cisgenderism as working at a micro-level by illuminating subjectivity and personal experiences (Serano, 2016). Ansara and Hegarty (2014) argue, however, that a more helpful perspective problematizes actions that marginalize trans or nonbinary people at all levels as cisgenderism operates on multiple levels, within individuals and communities as well as within institutions and social structures (Ansara & Hegarty, 2014; Rogers, 2017a, 2017b).

Cisgenderism can be examined as an ideological imperative that is systemic and prejudicial in nature. Operating at this macro-level, cisgenderism is akin to sexism and racism. It is prevalent and commonly reflected in authoritative cultural discourses (Ansara & Hegarty, 2014). Cisgenderism can be examined as an ideology that marginalizes and delegitimizes people’s self-identification in terms of gender and self-designation of the body as one thing or another. It is important to bear in mind that cisgenderism can be both intentional or unintentional (Ansara & Hegarty, 2011, 2014). Theories of normativity in relation to sexual identity are also relevant to the analysis because sexuality and gender are often conflated, particularly for minority groups, and it can prove difficult to partition experience in terms of the two (Rogers & Ahmed, 2017). Comparable to gender normativity, the concept of heteronormativity is relevant too as it privileges heterosexuality as the norm and positions homosexuality as deviant (Hall, 2010).

Cisgenderism is multidimensional and can operate through several modes of maltreatment and abuse. This includes practices of misgendering and pathologizing. Misgendering refers to the specious use of gendered language which does not reflect how a person self-identifies (Ansara & Hegarty, 2014). Examples of misgendering at a micro-level include using a person’s previous name or gender pronoun which does not accurately convey their self-designated gender. Misgendering also occurs at a macro-level when, for example, a system, structure, or norm (social, medical, economic, or legal) does not recognize a person’s self-identification or self-designated body. For example, in the United Kingdom someone may identify as a man or woman, but unless they have a Gender Recognition Certificate (applied for under the Gender Recognition Act 2004), they cannot apply for a replacement birth certificate in their self-identified gender.

For the purposes of the analysis here, misgendering is an active, not passive, act of abuse. Similarly, pathologizing refers to the intentional action of labeling and treatment of people’s self-identified gender, body, presentation, and practice as abnormal or deviant (Ansara & Hegarty, 2014; Riggs et al., 2015). Both misgendering and pathologizing are microaggressions which target a person’s gender identity or expression. Microaggressions are commonplace within everyday interactions and serve to denigrate and *other* people (Thurber & DiAngelo, 2018). Microaggressions permeate everyday reality for marginalized groups. Microaggressions can be verbal, nonverbal, and symbolic communications such as derogatory remarks or terminology, facial expressions, body language, or environmental slights that communicate hostility or offense (Sue, 2010). While modest, there is a small body of work exploring microaggressions experienced by trans people (Nadal et al., 2014; Pulice-Farow et al., 2017).

Within the discourse of DVA, cisgenderism has considerable potential to be influential as theoretical analyses and empirical studies have largely been rooted to a gender paradigm which positions men as perpetrators and women as victims (Rogers, 2017a). Donovan and Hester (2014) persuasively argue that this has resulted in the widespread acceptance of a “public story” of DVA as a problem of physical violence within heterosexual relationships. Historically, this has resulted in the neglect of people who exist outside of the “public story” including trans and nonbinary people (Cannon & Buttell, 2015; Rogers, 2017a). Kennedy (2013) would argue that the invisibility of trans people in statistical data, in particular, and discourses of DVA, in general, is due to the consequences of cultural cisgenderism. Thus, the authoritative cultural discourse (Ansara & Hegarty, 2011, p. 4) of DVA, the “public story” can be seen to invisibilize, silence, and invalidate trans and nonbinary people’s experiences as DVA is conceptualized as hetero- and gender normative; a systemic practice known as “trans-erasure” (Serano, 2007, p. 189).

## Research Methods

In this article, data are drawn from two qualitative empirical studies and re-examined. First, a doctoral study exploring trans people’s narratives of domestic abuse (including IPV and FV) was completed between 2010 and 2013 with ethical approval from the University of Sheffield, United Kingdom. This study sought to explore trans people’s experience of IPV and FV as well as their experiences of and perspectives on existing specialist support for people escaping domestic abuse. Second, data gathered for a program evaluation of a domestic abuse service provided by a U.K.-based LGBT organization was undertaken in 2016 to 2017. The study received ethical approval from the University of Salford. This evaluation centered on the following service-specific questions: To what extent has the service met its intended outcomes; how effective is the current service in terms of outcomes from the perspective of service users; and what impact has the service had on the lives of LGBT people living with or fleeing DVA in the city?

### Theoretical and Philosophical Influences

A constructionist ontology and feminist paradigm undergirded both studies. Ansara and Hegarty (2014) advocate embedding a framework constituted by the concepts of gender normativity and cisgenderism within a methodology as beneficial in feminist projects. They maintain that critical frameworks such as this have the potential to reduce discriminatory gendered ideology. The theoretical and empirical analyses benefit from an intersectional lens where appropriate (which views identity as multiple, dynamic, situated, and contingent) to illuminate the subjectivities and specificity of DVA for trans and nonbinary participants who have diverse backgrounds as well as embodying and practising different forms of trans and nonbinary gender (see Meyer, 2012, for a more detailed analysis of antiqueer violence using an intersectionality lens).

### Participants

Both studies used a purposive sampling strategy with the same two criteria regarding gender identity and experiences of domestic abuse (total sample *n* = 24). The first study (sample size *n* = 15) recruited via virtual chat rooms, trans organizations, and social/support groups. The evaluation (sample size *n* = 9) integrated inclusion criteria that required participants to have received support from the service. All participants identified across the gender spectrum as trans (e.g., trans man, trans woman, person with a transgender history, MtF, and FtM) and/or nonbinary (e.g., genderqueer and gender fluid). Of the total sample, 14 identified as women, 6 as men (at junctures on the gender spectrum), and 7 as nonbinary (some participants ascribed to multiple gender identities). The second study included people who identified as a sexual minority and cisgender, and subsequently their narratives are excluded from this analysis. Participants’ self-designated labels and preferred pronouns are used in the reporting of findings. All participants have been given a pseudonym and to ensure confidentiality and anonymity, any other potentially identifying information has been changed.

### Data Collection

The data collection method was similar across the studies as both employed semistructured interviewing. Interviews took place in accord with the participants’ preferences in terms of time and place with email or face-to-face interviews offered in the first study, and telephone or face-to-face interviews offered for the evaluation. All interviews were digitally recorded (with consent) and transcribed by the researcher. Due to the sensitive nature of the topic, all participants were provided with documentation detailing the project’s aims and research questions in order that consent was fully informed.

### Secondary Data Analysis

Data have been re-analyzed using thematic analysis (Braun & Clarke, 2006) to consider how participants described their individual experiences of IPV and FV in the context of cisgenderism and its enactment through identity abuse, microaggressions, misgendering, and pathologizing. Using Braun and Clarke’s method, data were coded to identify broader themes which are presented below in the findings section. A secondary analysis of data is now a widely recognized methodology which facilitates an extension of the analytical depth of the original work, a process termed by Thorne (1994) as *analytic expansion* (Corti et al., 2005; Rogers et al., 2019). Secondary narrative analysis serves to extend the initial analysis by returning to key themes within the context of new or contemporary theoretical frameworks (Elliot et al., 2015). In this instance, the re-analysis integrated the concept of cisgenderism and specific modes of abuse enacted as microaggressions. The narratives presented in the findings section are purposively selected to illustrate these different modes of cisgenderism and how they are enacted as IPV or FV.

### Researcher Positionality

In acknowledging the power and privilege within the researcher/researched relationship, as well as the researcher’s cisgender and heterosexual positionality, the following principles were adopted:

to engage in LGBTQ research is to embrace and question fluid identity positions and to be committed to openness. . . perhaps, most importantly researchers need to be self-reflexive, linking knowledge and understanding gained to action taken to give LGBTQ persons presence and place in education and other communities where they can be visible and proud, respected and valued. (Grace et al., 2006, p. 340)

These principles were adopted in addition to the researcher’s professional ethics as an experienced and registered social work practitioner and academic.

## Findings

Collectively the data highlight different forms of abuse, and findings are organized thematically to illustrate different forms of cisgenderism, namely: embodiment and identity abuse; intersectionality and identity abuse; misgendering; and pathologizing. In this article, “identity abuse” is a form of DVA that uses gender normative and cisgenderist ideas and beliefs to denigrate, coerce, and control (Woulfe & Goodman, 2021). An analysis of each person’s experience is undergirded by the theoretical and conceptual framework with a clear delineation of how the concepts of gender normativity and cisgenderism map onto experience.

### Embodiment and Identity Abuse

Understanding gender identity in relation to an embodiment paradigm requires a consideration of the body as a corporeal entity that is articulated and produced through socio-cultural processes (Pilcher & Whelehan, 2017). Such processes are imbued with norms that promote notions of what it is to be a man or a woman and how these ideas undergird normative conceptions linked to aesthetics, expression, and identities. For many participants, embodiment and bodily aesthetics were illuminated in their narratives of abuse. For example, in depicting her strained familial relationships, Rachel (21 years old, genderqueer) described her father’s actions in planning a family trip:

I’m convinced dad didn’t want me to go. . . . He didn’t book hold luggage even though he knows I need a range of clothes so that I can make sure I’m both comfortable myself and not generating tension.

Rachel’s father struggled to accept her cross-gender identity and presentation (reflecting dominant ideas of gender as binary and stable, rather than queer and fluid), yet for trans people, identity, the body, and embodiment are critical dimensions in the accomplishment of a gendered self. In Rachel’s narrative, her father’s actions appear to be motivated by the desired consequence of disrupting Rachel’s autonomy and capacity to express her cross-gender identity. Rachel experienced frequent microaggressions as identity abuse. Her father had previously asked, “Can’t you just be a gay man?,” concurrently indexing Rachel’s previous identity as well as different levels of acceptability in terms of sexual orientation and gender identity. Identity abuse results from an interplay of both micro- and macro-level dynamics in that broader structural forces (forms of systemic oppression such as sexism, racism, or ableism) are deployed to control, manipulate, or cause harm (Guadalupe-Diaz & Anthony, 2017; Woulfe & Goodman, 2021). The dynamics of identity abuse often target identity-based vulnerabilities (Brown, 2011) drawing on a combination of these wider ideologies, or honing in on one aspect of identity (see the next section for a discussion using intersectionality as a conceptual tool).

Another participant, Julie (62 years old, trans woman), described how her ex-wife (Mary) similarly targeted the everyday signifiers (clothing and accessories) that Julie needed to express her gender identity. Julie said:

So, if I wanted to buy, or bought, anything feminine for me, all hell would break loose as I’d be wasting money that should be spent on the family.

Mary controlled all the finances meaning that Julie had to request “spending money” if she needed anything. On one occasion, Julie used some of this money to buy a pair of tights. The next day, Julie found these destroyed and deposited in the rubbish bin. This pattern of incidents left Julie in a perpetual state of confusion as to whether Mary accepted or rejected her trans identity. The ways in which Mary controlled Julie’s expression of gender identity within the context of their family life reflects dominant narratives about the family as a system in which gender operates as an organizing principle and, at the time of this incident, Julie was designated as the “breadwinner” in the family. She had not transitioned at work; she was employed as school teacher, a role she performed in her previous male identity. Highlighting the ways that dominant ideologies about institutions, such as the family, can influence individual autonomy, decision-making, and actions, when it was convenient, Mary drew from the “male breadwinner” trope to justify her actions.

Through his childhood, Ally (27, trans male) experienced considerable psychological discomfort in relation to his physical body which had led to mental health problems needing therapeutic intervention. Ally described his father’s response to his disclosure that he was trans and male as one that negated Ally’s trans identity and had other health implications:

My therapist, whom I had just recently started seeing for general depression, and who had recently diagnosed me with [Gender Identity Disorder] without explaining it in a way I could understand, let me read my dad a letter in her office. [. . .] That night he got so drunk he could barely talk. He said I would always be his daughter and he would always love me, but he [. . .] wouldn’t speak to me for a month. And then after that, it was something we were supposed to never talk about again, and I was supposed to prove that I was a worthwhile *female* human being. Oh, and he told me, “Well, since that’s over with, I guess you don’t need to go to therapy anymore, huh.” *Just* when I had been diagnosed with [Gender Identity Disorder], which is certainly why I was so depressed and bordering on psychotic in the first place.

The denial of Ally’s self-ascribed gender identity by his father clearly illustrates the operation of gender normativity. Like Ally, another participant, Max (25 years old, trans/femme male), also experienced poor mental health at times. His experiences of identity abuse were plentiful with some ex-partners building on his insecurity around bodily aesthetics as he admitted that “I struggle to not compare my body in an unfavorable way to the bodies of cis men.” When Max disclosed his trans status to his then partner, Su, she responded in a negative and unsupportive way. Su exploited embodiment narratives that drew parallels with male bodies as representing a violent threat to women’s safety and well-being. Max explained:

My partner at the time was honest with me about the fact that she doesn’t feel as safe around men or around “male” bodies, and that she may no longer feel safe around me as my physical transition progressed.

Su’s response signifies the norms that are systemic and rooted in notions of hegemonic masculinity and gendered embodiment (invoking the male as dominant/female as subordinate dichotomy) (Connell, 2005). The ways that these norms underpinned Su’s agency (power to make decisions and moderate actions) in her intimate relationship with Max also reflect cisgenderist constructs. Su’s perspective and actions had considerable impacts for Max as he noted that “this fed into my already existing fear that my masculinity may be experienced as dominating or intimidating.” Yet, Su’s actions were not based on Max’s physical body or presentation, as he added that “I was so far from presenting as male at the time,” and he described his physique as “small and femme.” Su’s actions were rooted in ideological mechanisms embedded in social systems and structures that reflected gender normativity and notions of hegemonic masculinity.

In addition, Max interpreted Su’s actions as tactical having the intention of undermining his gender identity and stalling the transitioning process. Gender transitioning is the process by which a person seeks gender affirmation by legal, medical, and/or social processes and begins to live as a gender that is different from their sex assigned at birth. As such, Max said, “I held back on expressing my masculinity around her, because I was afraid it would be triggering for her.”

Ally also described how a former partner used the notion of “being triggered” as a way to denigrate his gender identity. Ally said, “Several times she told me I was triggering her because she hated men.” Ally’s partner identified as “MTF and genderqueer/nonbinary,” but this commonality in having a gender identity different to that which was assigned at birth did not generate empathy nor support for Ally’s desire to transition. It had a converse effect as Ally described:

I was also hesitant to start taking testosterone because she referred to her time (unwillingly) on testosterone with such disgust, I was worried that she would find me repulsive. But at the same time, she also told me once, to do whatever steps in transition I needed to do, without worrying about what she or anyone else thought.

For Max, there were social and relational consequences of his experiences that underpinned the way he felt about future partners and relationships:

Dating and hooking up is a whole other issue. . . . The main issue for me is disclosure. It’s hard to know how safe it is to come out to someone. . . . I’ve been worried that they would misgender my body, and not treat me as male, which I would find deeply traumatising. Regarding the people who have wanted to have sex with me without knowing that I’m trans, I haven’t considered going for that option because I am fearful of the negative and potentially violent and/or abusive reactions I may experience when the person finds out I’m trans.

Moving away from microaggressions to overt displays of physical violence, the narrative of Joe (46 years old, trans male) highlighted identity abuse and embodiment as a cultural process but one that was tied to essentialist ideas of binary gender as fixed and immutable. Joe’s partner, John, denied Joe’s changing gender, and the excerpt below illuminates an instance when identity abuse and physical violence overlapped. Joe said:

John turned and grabbed my shirt and, right in my face, shouted, “You’ll never do it. You’re not a man. I’m a real man. You’re a fucking woman, [Joe]. You will always be a woman. A woman with a vagina to have sex like a man and woman should [. . .] You can wear what you like, talk like a man. You will *always be a woman*.” His face was so close to mine. I thought he was going to attack me.

During another incident, John grabbed Joe’s breasts and said “real men don’t have these.” The adoption of gender normative beliefs (incorporating ideas about male and female identity being fixed to particular sexed bodies and sexual practices) appears to strongly undergird John’s behavior in these incidents.

## Intersectionality and Identity Abuse

Some participants illustrated the intersections of gender, trans, and other identity categories (such as sexuality, ethnicity, and age) in their abuse narratives. An intersectional lens, similar to that applied by Meyer (2012) in his analysis of antiqueer violence, illustrates the complex multiplicity of identity in relation to abuse experiences. For example, Ally came from a family background with a mixed heritage; his mother was white British, and his father was Asian. Ally’s experiences of FV highlighted the powerful intersection of gender, sexuality, and cultural norms, as macro-level structures of social control and organization, operating at a micro-level within personal relationships. Ally also experienced IPV. During adolescence, Ally had identified as a lesbian, before being diagnosed with Gender Identity Disorder (GID). After disclosing his trans status to his family and girlfriend at the time, Dee, Ally began to adopt practices and signifiers (clothes and hairstyle) typically associated with masculinity to signify his male identity. At first, Dee was supportive of his decision to transition, but this support waned. Dee began to make derisive comments about Ally’s trans status and his increasingly male presentation. The latter would be used in a way to undermine his identity, relationships, and cultural belonging with references to his family dynamic and community background:

She’d say, “Are you doing this to further piss your dad off? His good [Asian] daughter, turns out to be a lesbian, and now you’re wanting to look like a man, like a son?” And saying that my dad would reject me, and our community would too.

This illustrates the multifaceted nature of identity as well as the complex implications of wider cultural beliefs, norms, and discourse. The reference to rejection by Ally’s family and community is an example of this in terms of cultural influences that are used within personal or intimate relations in an abuse of power and control.

Several participants linked their personal experiences of abuse to gender and the intersection of disability and health. Rachel conflated her experiences of identity abuse in relation to her gender and her identity as an autistic person, highlighting the systemic influences of normativity (both cisgenderist and ableist) as undergirding her family’s norms and actions. Offering a different perspective, Ally described his mental ill-health as resulting from his experiences of frequent microaggressions and living in a perpetual state of tension and confusion. Poor mental health as a negative outcome is a common result of living with abuse, as there is ample testimonial and empirical evidence portraying the deleterious impacts of microaggressions (Thurber & DiAngelo, 2018).

## Misgendering as a Form of Cisgenderism

For trans people, vulnerability can be greater when they are in the process of transitioning. Jane (42 years old, trans woman) described her mother’s persistent denial of her gender identity and physical transitioning:

She’d see my nails getting longer. See my hair getting longer. Sometimes I actually went around with a dirty great blob of red on my neck or face where I’d had electrolysis. She thought it was a razor rash at first and then she said, “What is it about your neck?” and I said, “I’ve had electrolysis.” She’d also seen I was wearing female jeans; denim was thinner and softer. I went around one day wearing this sort of shoe (women’s pumps). So now she’d got the tights, the shoes, the nails. She’s got the hair [. . .] She’s seen me in my full silver service uniform of skirt, buttoned female waistcoat, bright red nails to die for. I’ve got a name badge that says [Jane] which I don’t hide [. . .] and she still calls me [David].

Jim (30 years old, trans man) described similar experiences of misgendering from his ex-partner, Emma. Emma’s reaction when he disclosed his trans status was to actively deny Jim’s male identity as she said, “It’s shit. I don’t believe you.” Jim and Emma had previously been in a lesbian relationship for several years. They separated 7 months after Jim came out as trans. Throughout those 7 months, Emma misgendered Jim directly in their interactions but also to other people, as Jim noted:

She’d talk about me to others and say, “Have you heard what [Paula] is claiming? *She’s* saying *She’s* identifying as a man now.” [Emma] refused to call me [Jim].

Emma refused to use Jim’s chosen male name or male pronouns and insisted on using Jim’s former female name and female pronouns. In doing so, Emma invalidated Jim’s agency as an individual and drew on normative gender as a means of social recognition. This resulted in her denial and rejection of Jim’s gender identity as a trans male. As shown in the quote above, Emma frequently emphasized the words that she used to signify the female identity that she continued to ascribe to Jim, which Jim experienced as a microaggression and as abusive. Mo (aged 23 years, trans male) had a similar experience after disclosing to his former partner, Kate, that he was trans, as she frequently used his female name and female pronouns. Mo described another tactic of identity abuse as Kate made frequent threats to out him to his colleagues in the school where he taught (outing is another form of identity abuse). In this way, Kate’s behavior was incoherent; on one hand, she would employ misgendering as a device of abuse and one that resulted in negating Mo’s identity, but concurrently she would threaten to out Mo in his self-designated gender.

The experience of misgendering described by Caroline (63 years old, woman with a transsexual history) was overtly active, as opposed to passive, as illustrated in the following excerpt:

I’ve a brother who doesn’t acknowledge me. Well, he acknowledges me in the fact that we exchange cards [. . .] That’s the one bad thing that’s happened in my life [. . .] they buy predominantly male-oriented birthday cards, Christmas cards with pint pots and footballers on and that.

Even in the limited contact that Caroline had with her brother over the years, he was steadfast in his refusal to accept her female gender identity, emphasizing this denial in his communication through intentional misgendering.

## Cisgenderism in Pathologizing Practices

Another common form of cisgenderism found within the narratives was the practice of pathologizing. Pathologizing, in relation to trans and nonbinary gender, refers to the process of designating or treating a person’s gender, body, expression, and experiences associated with their gender as defective (Riggs et al., 2015). Pathologizing operates in terminology that is grounded in medical discourse around trans-related issues and employs negative classifications such as GID and *gender dysphoria*. Ally’s narrative offers examples of a family’s response to someone who has been diagnosed with GID:

Lots of emotional abuse seeped out when I came out to my parents as trans [. . .] My dad of course made it sound like I would be a shame to all of my [Asian] family, and all of his friends. My mom told me she knew I was “gender confused” but thought I would grow out of it, and suggested that I go for restorative therapy.

To contextualize Ally’s experience, it is useful to apply a framework which considers cisgenderism as a prejudicial attitude that contributes to unequal gender relations which, in turn, undergirds a gender hierarchy (Connell, 2005; Stryker & Aizura, 2013). This operates to position trans-identified people as *abnormal* and as *other* (Serano, 2016; Stryker & Aizura, 2013) (with *othering* representing another form of cisgenderism). Rachel’s family also used *othering* devices to deal with her queerness and genderqueer identity. Rachel depicted the negative attitudes that frequently underpinned her stepmother and birth mother’s behavior toward her:

[My step mother] is particularly prone to passive-aggressive insults, especially about my apparent failure to be normal. Mum’s version of this is to tell me how much harder life is when I’m around. I’ve been staying with Mum [. . .] and she comes out with all these belittling remarks about my psychiatric impairments.

Rachel experienced her family’s pathologizing as a way to position her on the edge of their family. Serano (2016) contends that such hierarchies sustain cisgender privilege (the power and privilege that cis people enjoy over trans people due to the influence and workings of gender normativity at both micro and macro levels). Participants depicted this privilege in their narratives, with one person describing his abuser as taking “the moral high ground.” It is the entrenched, daily workings of gender normativity and cisgender privilege that underpin the configuration of beliefs and attitudes toward trans people in this regard (Serano, 2007, 2016). This is because while cisgenderist ideology remains dominant and is characterized by the social categories of men/masculine and women/feminine (the normative framework for understanding gender), a system of power and privilege is maintained that subjugates trans identities, expressions, and experiences (Ansara and Hegarty, 2011, 2014).

Both Caroline and Julie had experienced rejection by their brother because of their trans identity. Julie described her brother’s rejection as well as other family members’ difficulty in accepting her:

My brother—I have one brother—was OK in the beginning but then he found it embarrassing and very shameful [. . .] My auntie couldn’t handle it very well. My cousin, [Helen], was dreadful. [. . .] I didn’t realise that the whole of my extended family cut contact with me [. . .] Only [Anthony] has kept in contact—secretly—so that was the end of my wider, extended family.

Anthony identified as a gay male and Julie had questioned his acceptance within the family in contrast to her rejection. This draws attention to the different levels of acceptability in relation to sexual orientation and gender identity within a sexuality and gender hierarchy where trans and nonbinary are positioned at the bottom (cisgenderism in operation) (Connell, 2005; Stryker & Aizura, 2013). This is also depicted in the behavior and language choice of Mary, Julie’s ex-wife, who used negative and pathologizing language when talking about the LGBT people who Julie was connecting with for support and friendship:

saying all these queers and crazy people [. . .] scum of the earth and all that kind of thing [. . .] My friends, or anybody, she’d use the most foul language to rubbish them . . . anybody who I’d be involved with outside of the family would be absolutely rubbished and trashed and she’d use the most violent language possible.

Pathologizing actions can result in stigma, marginalization, and social isolation. Joe’s partner, John, struggled to accept his partner’s disclosure as trans and adopted a means of understanding this by way of a medical model. Joe explained:

John couldn’t get his head round it. In the first few months he kept telling me to get to the doctors and that I obviously had something wrong with my head. I told him something had always been wrong. I always hated my body. He knew that. So, then he said that I was mentally ill and that hating my body was part of it. We hadn’t had sex for about two years, and he brought that up and said it was my fault because of my body. He used it all against me.

In this excerpt, John pathologizes Joe’s identity and his psychology but also draws on this and their lack of intimacy, laying the blame with Joe for all that was wrong within their relationship.

## Discussion

The findings presented illuminate the extensive ways in which cisgenderism can be operationalized through the practices of partners and family members within intimate and familial relationships with trans people. Not only were these abuses multiple in their expression, but the various microaggressions described by participants were experienced frequently, both in private and more openly, in front of others, and always as active, not passive or unintended, actions. All forms of cisgenderism and microaggressions had significant impacts for the participants, and in addition to stigma and isolation, there were reports of internalized transphobia, poor mental health, physical ill health, social isolation, and relationship breakdowns. Moreover, research has identified that family-level microaggressions are a risk factor for polyvictimization as well as the negative outcomes listed here (Sterzing et al., 2017).

Participants spoke about the emotional and psychological impacts of rejection and being belittled, dismissed, and invalidated. The notion of invisibilization was a recurring theme in which participants felt invisible in their own home and social networks. In her persuasive treatise, Serano (2007, p. 189) labels this as “trans-erasure”; a process that marginalizes and negates the very existence of trans people. The sensation of invisibilization, or trans-erasure, reflects the workings of cisgenderism as an ideology that disempowers and de-legitimizes people in terms of agency and the ability to self-identify as one gender or another. Therefore, the embodiment narratives of participants were often imbued with contestation and tension as those frequent cisgenderist microaggressions resulted in troubling people’s confidence and ease in enacting their gender identity or moving through the transitioning period. Moreover, the data illuminate forms of cisgenderism, in addition to misgendering and pathologizing, as being *marginalizing* and *invisibilizing* (or *erasing*).

A further example of invisibilization through the operation of a gender hierarchy concerns the way in which trans people can be neglected within dominant and influential discourses, a phenomenon Rogers (2016a, 2017a) explores in her study on IPV and FV. This invisibilization is problematic, as it leads to the neglect of marginalized groups within research, policy, and practice and extends the “public story” of DVA (Donovan & Hester, 2014). This neglect results in the limiting and channeling of the allocation of resources and pathways for support. As such, it is unsurprising that trans-identified victims-survivors exist outside of the public story and experience many barriers to accessing formal support (Cannon & Buttell, 2015; Rogers, 2016a). In addition, at a micro-level, this absence or neglect in dominant discourse results in myopic understandings of what constitutes DVA with individuals unable to recognize and name their experiences as abuse.

This analysis illuminates how microaggressions, when experienced by trans people, are identity sensitive (Pulice-Farow et al., 2017). Some participants spoke about their experiences of everyday cisgenderism as identity abuse in the context of their desire and ability to *pass*. Begun and Kattari (2016) describe passing as the process which enables a person to be recognized, or pass, in their self-identified gender. Passing can be linked to experiences of cisgenderism when those cisgender-based norms that influence notions of bodily aesthetics and expression provide a framework by which to judge someone’s presentation (Gagné & Tewksbury, 1998; Rogers, 2017a, 2017b). The concept of passing is contested, however, when it relies on cisgender-based norms, which are reified through aesthetics and practices, and passing, therefore, is not significant nor desired by people who identify as nonbinary (Bornstein, 2016). Notwithstanding, for those participants who did identify within the gender binary, microaggressions were sometimes tied to a person’s incongruent aesthetics in terms of normative gender signifiers (for example, height and body frame designated as masculine/feminine, facial hair designated as typically masculine). In some instances, maltreatment resulted in participants moderating or ceasing from expressing their gender identity, an intended outcome of trans-erasure (Serano, 2016).

It is important to acknowledge that there are additional aspects of social identity, in addition to gender, and that participants do not experience life as a gendered person in isolation from other social characteristics. As such, a one-dimensional analysis of identity is problematic. Moreover, theorizing the interconnections between identity and marginalization in this way inescapably fails to account for the ways in which forms of discrimination or abuse, such as those based on gender, ethnicity, or class, interrelate (Crenshaw, 1989; Hines, 2011). Intersectionality scholars have been instrumental in illuminating the systematic means by which such forms interlock and shape each other (Hines, 2011). There is an emerging body of work detailing trans people’s experiences using an intersectionality lens but in relation to trans masculinity, in particular, this literature tends to reflect the dominant white, western-centric contexts in which they have emerged (Rogers, 2020; D. Valentine, 2007).

The emerging literature has progressed understandings by emphasizing the social, cultural, and spatial variations of possibilities for trans expression (Gordon & Pratma, 2017), and there are some examples of intersecting elements of identity integrated earlier in this article with attention given to the convergences of gender, sex, mental health, and ethnicity. In a systematic literature review that sought to examine IPV in ethnic minority LGBT populations, West (2012) highlighted the lack of empirical studies to-date, although she draws attention to the higher rates of IPV for same-sex couples. West simultaneously exposes the overwhelming knowledge gap for ethnic minority trans people illuminated through an examination of extant studies that purport to include a trans perspective. In addition, the knowledge gap in relation to trans people’s subjective experiences of DVA results from the entrenched nature of cisgenderism. The value of employing an intersectionality framework is that when scrutinizing trans people’s narratives of abuse and maltreatment, it can serve as a reminder that lived experiences are often correlated with multiple, not singular, aspects of a person’s identity and social location.

## Conclusion

Cisgenderism is pervasive. It operates as an intentional or unintentional strategy, ideology, and/or force to influence people’s everyday behaviors and experiences on one level, and institutions and structures on another (Ansara & Hegarty, 2014). As such, it is a complex phenomenon operating at different levels, but one thing is evident: When it is operationalized through people’s actions, it can result in poor outcomes for people who do not identify within normative conceptions of gender. In this article, the data presented have been analyzed within a framework which articulates gender normativity and cisgenderism as conceptual tools to understand the ways in which DVA (IPV and FV) is experienced by trans and nonbinary people. This analysis illustrates how cisgenderism is enacted through microaggressions in different modes, as identity abuse in relation to embodiment processes, and through misgendering or pathologizing practices.

Finally, there is a dearth of empirical work which explores trans people’s intimate and familial relationships and environments in relation to cisgenderism. Yet, more broadly, existing studies indicate that LGBT people are at a high risk of IPV or FV victimization and trans people may be at even higher risk than their cis LGB peers (Langenderfer-Magruder et al., 2016; S. E. Valentine et al., 2017). The value of this article then is that it contributes to the extant empirical and theoretical scholarship that examines trans and nonbinary people’s experiences of abuse and microaggressions within intimate and familial relationships. The article highlights the need for further research that robustly addresses the continued invisibilization of trans people within the discourses of IPV and FV. It adds to the emerging literature that employs cisgenderism as a conceptual tool to understand trans people’s experiences of marginalization, social isolation, and abuse (Rogers, 2017a, 2017b, 2020). This is significant, as there is a mounting evidence base detailing cisgenderism and/or microaggressions in other areas of personal life, such as within friendships and in educational experiences (Ashley et al., 2016; Pulice-Farow et al., 2017). Without further work, it is likely that research, policy, and practice pertaining to DVA will remain rooted in dominant discourses and the public story (Donovan & Hester, 2014) and, as such, this article calls for further scholarship in this field using cisgenderism as a conceptual tool to examine DVA, in all its forms, in the lives of trans people.
